# Remote sensing of Pu in uranyl nitrate crystals using reflectance spectroscopy and chemometrics†

**DOI:** 10.1039/d5ra08550k

**Published:** 2025-12-09

**Authors:** Luke R. Sadergaski, Jeffrey D. Einkauf, Laetitia H. Delmau, Jonathan D. Burns

**Affiliations:** a Oak Ridge National Laboratory Oak Ridge Tennessee USA sadergaskilr@ornl.gov; b Department of Chemistry, University of Alabama at Birmingham Birmingham AL USA burnsjon@uab.edu

## Abstract

Remote quantification of Pu(vi) (0–5 mol%) co-crystallized with U in uranyl nitrate hexahydrate (UNH) crystals was achieved in a glove box using reflectance spectroscopy coupled with chemometric modeling. Reflectance spectra were also acquired for Pu(iv) and Np(vi) (0–5 mol%) crystallized with UNH; revealing spectral features consistent with their solution-phase analogs. Principal component analysis revealed Pu(iv/vi) and Np(vi) concentrations as the primary source of variation in the data, informing the development of a supervised partial least squares regression model for Pu(vi). The resulting calibration demonstrated robust performance, with replicate root mean square errors near 10% and quantifiable limits near 0.2 mol% Pu(vi) relative to U. The Pu(vi) remained stable in the crystalline UNH matrix for at least one week with minimal reduction to Pu(iv). Notably, Pu(vi) and Np(vi) incorporation in UNH quenched U(vi) fluorescence while Pu(iv) did not. This study presents a noninvasive, spectroscopic approach for solid-state Pu quantification, with direct implications for material accountability and nuclear nonproliferation monitoring.

## Abstract 2

Remote quantification of Pu(vi) (0–5 mol%) co-crystallized with U in uranyl nitrate hexahydrate (UNH) crystals was achieved in a glove box using reflectance spectroscopy coupled with chemometric modeling.

## Graphical abstract

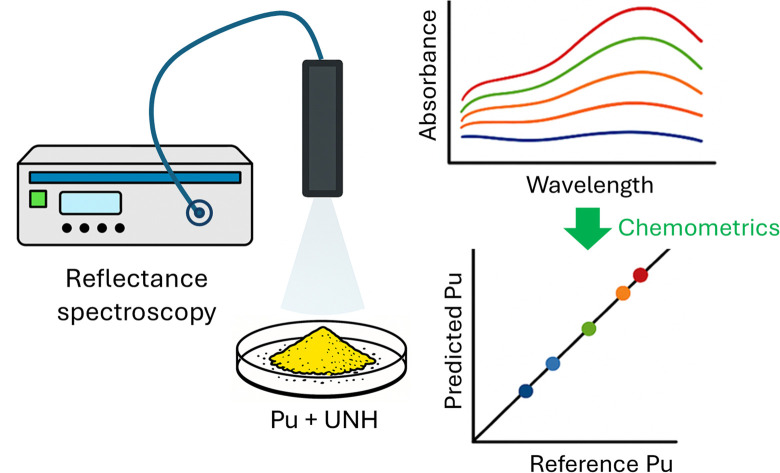

## Introduction

Optical spectroscopy and chemometric techniques are being developed for chemical process monitoring and material management purposes in the nuclear field.^1,2^ The need for robust in-line monitoring strategies is expected to grow with increasing adoption of nuclear energy, a promising technology that can help address increasing global demand for energy and reduce carbon emissions.^3^ Advanced, proliferation-resistant actinide separations are needed to help close the nuclear fuel cycle to bolster nuclear energy and minimize the nuclear waste stored in repositories.^4–7^ Online monitoring with spectroscopy is a valuable tool for monitoring, tracking, and improving radiochemical separations. Most applications in various aspects of the fuel cycle are primarily focused on the quantitative analysis of liquid samples using UV-Vis-NIR absorption or vibrational spectroscopy (IR and Raman).^1^ Such efforts will benefit from continued expansion into additional optical techniques for the analysis of diverse material types (*e.g.*, solids).^8–14^

For several decades, reflectance spectroscopy (RS) has been applied for solid materials characterization and quantification purposes in process environments (*e.g.*, food, biomedical, pharmaceutical) and as a robust means to measure soil properties in agricultural and environmental applications.^15–21^ Specular reflection generally carries limited analytical value due to minimal sample interaction and reduced absorption likelihood. By contrast, diffuse reflectance, arising from internal scattering, contains substance-specific spectral features and is preferred for quantitative analysis. Although internal reflectance (attenuated total reflectance, ATR) can analyze opaque samples often encountered with powders, the technique is most effective for relatively short-distance measurements up to approximately 1.5 m, so it cannot be deployed for noncontact measurements in harsh and remote environments.^22^ Raman spectroscopy has been used to characterize actinide materials but generally requires long integration times, and coupled interactions in crystal lattice vibrations render quantitative measurements challenging.^11^

RS is flexible, inexpensive, nondestructive, rapid, and amenable to in-line measurements using fibre optic cables. It is a promising option for the quantification of actinide elements in solid-state samples, yet its application in the nuclear domain is limited. RS may be particularly relevant for monitoring emerging reprocessing technologies, such as a transformational concept based on the co-crystallization of oxidized actinides with uranyl nitrate hexahydrate (UNH).^5,6^ This technology has the potential to recover oxidized forms of Np, Pu, and Am as actinyl ions NpO_2_^2+^, PuO_2_^2+^, and AmO_2_^2+^ within the solid UNH matrix while rejecting a majority of corrosion and fission products.^7^ Such a process would require analytical measurements for material control and accountability for Np, Pu, and Am in the UNH; these measurements could be accomplished using RS by quantifying material absorption features.^23–25^ Although radiometric techniques (*e.g.*, gamma spectroscopy) can be used to quantify Np and Am directly in solid-state matrices, Pu does not have intrinsic gamma signals and normally requires grab samples for measurements using offline techniques such as alpha spectroscopy, liquid scintillation counting, and mass spectrometry.^26–29^ RS has the potential for in-line quantification of metal ions in solid matrices and is particularly effective when combined with chemometrics.^10^

Complex, nonlinear and potentially convoluted spectral features common in reflectance spectra must be accounted for.^30,31^ Unsupervised and supervised chemometric techniques can be used to describe complex spectral features by finding patterns in the data and correlating spectra to concentration.^32^ Partial least squares regression (PLSR) is a supervised technique for relating *X* (*i.e.*, spectra) and *Y* (*i.e.*, concentration) data matrices in systems with complicated spectral features.^33–35^ PLSR has been used for decades with success in numerous fields.

Here, we demonstrate RS for the quantification of Pu(vi) in UNH crystals by PLSR in a glove box using fibre optics and a noncontact reflection probe. This work includes several points of scientific advancement: (1) reporting Vis-NIR absorption spectra of Np(vi), Pu(iv), and Pu(vi) ions crystallized in UNH matrices, (2) describing U(vi) fluorescence quenching when Pu(vi) and Np(vi) are co-crystallized in a UNH matrix, and (3) demonstrating how multivariate analysis can quantify relative Pu(vi) concentrations in UNH by RS in a remote environment. Combining RS and chemometrics has the potential for robust and rapid Pu measurements in solid-state samples to support diverse applications, including radiochemical process operations, safeguards, and material accountability.

## Methods and materials

### General materials

Concentrated HNO_3_ (70%) and ACS-grade NaBiO_3_ were purchased from Sigma-Aldrich. All samples were prepared using deionized water with a resistivity of 18.2 MΩ cm. Samples were prepared using calibrated pipettes. The UNH (99.9%) sample was purchased from International Bio-Analytical Industries, Inc. Both Np-237 and Pu-240 samples were prepared in-house at Oak Ridge National Laboratory. Sample compositions were confirmed by alpha spectroscopy (Canberra Alpha Spectrometer Model 7401) and inductively coupled plasma mass spectrometry (iCAP Q ICP-MS, Thermo Fischer Scientific). Warning: Np-237 and Pu-240 are highly radioactive and were handled under ALARA principles in laboratories equipped to handle radioactive materials appropriately. Radiological glove boxes were employed.

### Crystallization and sample preparation

UNH crystals were crystallized by heating a supersaturated U solution in approximately 3 M HNO_3_ to 58 °C and then cooled to room temperature, near 21 °C. Before the crystallization process, Pu-240 and Np-237 stocks were added to UNH samples using a volumetric pipette. Sodium bismuthate (NaBiO_3_) was added to oxidize Np and Pu to Np(vi) and Pu(vi). Approximately 100 mg of U in each sample was prepared in 20 mL glass scintillation vials. The crystals appeared to be translucent and ranged from bright yellow-green to a dark green with increasing Pu content or brown with increasing Np(vi) content.

To promote reproducible diffuse RS, samples were ground and packed to enhance homogeneity, as smaller particles increase interfacial area per unit mass. Preparation (*i.e.*, grinding and pressing) was intentionally minimal to accommodate operational constraints and assess performance under nonideal scattering conditions and minimize the possibility of exposing Pu(vi) to the environment, which could result in reduction to Pu(iv). Although particle size was not quantified, visual inspection suggested dimensions were above the measurement wavelength range (400–1100 nm), reducing wavelength-dependent scattering effects. However, absorption spectra were likely still influenced by heterogeneity and specular contributions. Samples were packed by hand with a pressing tool to a consistent thickness of around 5 mm.^30^

### Absorption spectroscopy

An Ocean Insight QEPro spectrophotometer was used to acquire reflectance spectra from 315 to 1110 nm at 0.77 nm increments with a 100 ms integration time and a five-scan average. Spectra were acquired using OceanView 2.0 software (Ocean Optics). An SL2 Mercury Argon Calibration Lamp (Stellarnet) was used to calibrate the instrument and determine a resolution of approximately 2 nm. A fibre-coupled Xe light source (SLS205, Thorlabs) and a stabilized incoherent light source (SLS201L, ThorLabs) were transmitted through several meters of multimode optical fibre patch cables into the glove box. Light from the sources was incident on the sample with a spot size of about 1 cm. A diffuse reflection probe (Ocean Optics) was used to acquire light undergoing reflection (both specular and diffuse) and transmit it *via* fibre optics back to the detector located underneath the glove box. The probe was held at approximately a 20° angle using a ring stand to help limit specular reflection. For most samples with rough or scattering surfaces, a fibre optic probe angled at 20° from the sample primarily collects diffuse reflections. Specular reflections are highly directional, so unless samples are very smooth, most of the measured light will be diffusely scattered rather than specular. Spectra were collected at numerous locations within each sample to provide a more representative average and to evaluate homogeneity. An Avantes WS-2 reflectance tile was used to reference the instrument. Reflectance spectra were recorded in absorbance units. Using absorbance units provides a more linear relationship with concentration, enhancing the detection of subtle spectral features and enabling more precise quantitative analysis. Additionally, since many established spectroscopic analyses and reference libraries are based on absorbance measurements, this approach facilitates easier comparison with published data.

### Chemometrics

The Unscrambler X (10.4) by Camo Analytics was used for principal component analysis (PCA), PLSR modelling, and preprocessing. Spectra were mean centered for PCA and PLSR. PLS1 models, which model one Y variable at a time, were created for Pu(vi), Pu(iv), and Np(vi). However, the performance of the PLS1 model for Pu(vi) will be the primary focus because it is the primary analyte of interest. Spectra were pre-processed using Savitzky–Golay (SG) smoothing, derivatives, standard normal variate (SNV), and de-trending to account for baseline offsets and other spectral artifacts. An SG filter with a first-order polynomial and three left/right points (seven total) was used for smoothing. SNV transforms each spectrum by subtracting its mean and then dividing by its standard deviation to correct for additive and multiplicative scatter effects. OriginPro was used for the Voigt function peak fit analysis.

For PLSR model development, replicate spectra from various sample locations were averaged to three representative spectra per sample, minimizing random noise and enhancing signal fidelity. A leave-one-out cross validation (CV) strategy was used to estimate the ability of the model to predict new, unseen data. The averaging approach ensured that the calibration set reflected the chemical variability across samples without artificially inflating the dataset. To evaluate model performance, the individual replicate spectra, excluded from calibration, were used as a validation set to assess predictive accuracy under realistic measurement conditions. This helped capture intra-sample variability caused by probe repositioning, optical scattering, and glovebox constraints. Although replicates are not fully independent, their use in validation provides a practical and informative estimate of model generalizability in constrained experimental environments.

### Statistical comparison

PLSR performance was evaluated using calibration, CV, replicate, and prediction statistics. RMSE of the calibration (RMSEC) and RMSE of the CV (RMSECV) were compared to determine whether the models were balanced, and RMSE of the replicates (RMSER) was used to assess repeatability. The true predictive power was evaluated using prediction statistics, including RMSE of the prediction (RMSEP) and percent RMSEP (RMSEP%). RMSEs were calculated using eqn (1):1
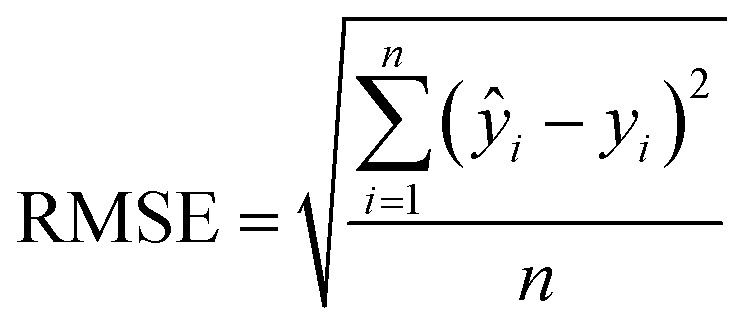
where *ŷ*_*i*_ is the predicted concentration, *y*_*i*_ is the measured concentration, and *n* is the number of samples. The RMSE% was calculated by dividing the RMSE by the midpoint of *Y* matrix values using eqn (2):2
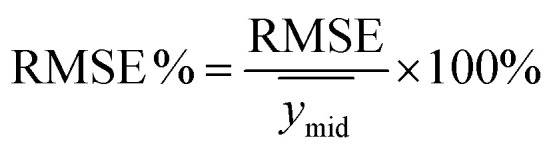
where 
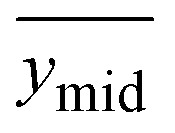
 represents the midpoint reference value. RMSE values are reported in analyte units. Evaluating replicate performance can be informative in spectroscopic studies where repeatability and precision are important. RMSE% values of ≤10% indicate acceptable performance.^36^

## Results and discussion

### Reflectance spectra

The UV-Vis-NIR electronic absorption spectra of U, Np, and Pu are governed by 5f–5f and 5f–6d transitions, which are sensitive to oxidation state, coordination environment, and ligand field effects.^24^ In the UV-vis region, fundamental electronic absorption bands dominate the spectrum; transitions for both Np(vi) and Pu(vi) species can also be observed in the NIR region (∼800–1250 nm). Both Np and Pu display rich spectral profiles across multiple oxidation states, resulting in overlapping transitions and complexity in mixed-valence systems.

**Fig. 1:**
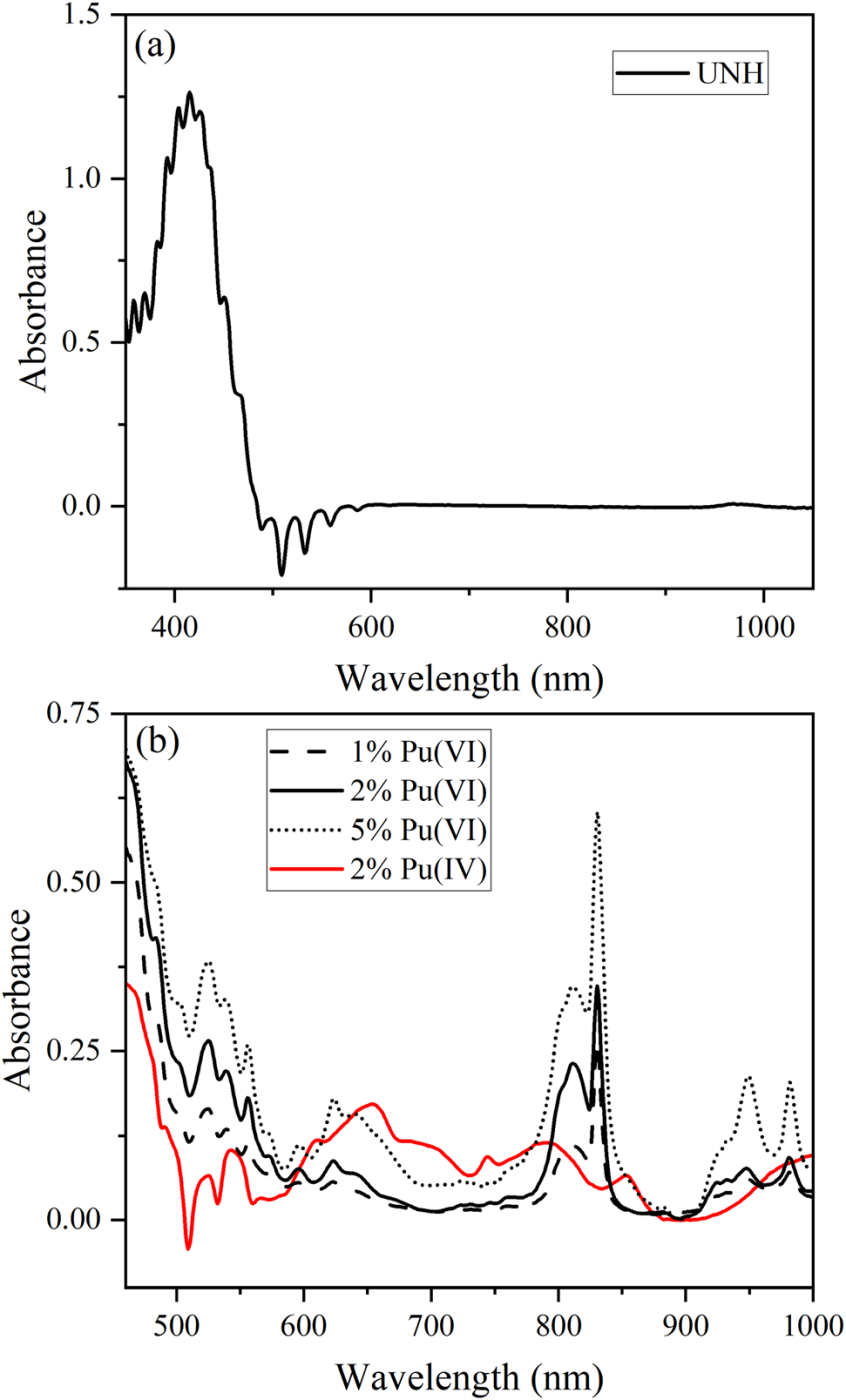
Reflectance spectra for (a) UNH and (b) 1, 2 and 5 mol% Pu(vi) or 2 mol% Pu(iv) in UNH. Note the different wavelength scales in (a) and (b).

RS measures the UV-Vis-NIR absorption spectrum of solid-state materials by measuring the intensity of reflected light as a function of wavelength. The intensity of the reflected light decreases at wavelengths where the material has characteristic absorption features. The Vis-NIR reflectance spectrum for UNH with 1, 2 or 5 mol% Pu(vi) co-crystallized or 2 mol% Pu(iv) precipitated with UNH is shown in Fig. 1. The characteristic ‘comb-like’ charge–transfer absorption and fluorescence U(vi) peaks were observed for UNH (Fig. 1a).^37^ Absorption peaks for UNH were located at 358.1, 369.3, 382.9, 392.4, 403.5, 415.4, 425.7, 436.7, and 450.2 nm, and fluorescence peaks (inverted) occurred near 488.7, 509.8, 533.8, 559.1, and 588.2 nm. The fluorescence peaks increased in intensity compared to the reference spectrum, resulting in the negative peaks. The illumination from a broadband light source caused U(vi) fluorescence. To avoid interference from convoluted U(vi) fluorescence features, future implementations could benefit in the 480–600 nm range from tailoring the light source to emit above approximately 460 nm, thereby minimizing fluorescence. The U(vi) absorption and fluorescence features correspond to vibrational levels within the electronic transition.^38^ The vibronic levels were relatively evenly spaced: the average difference between absorption levels was 11.5 nm. Beyond about 600 nm, the UNH spectrum baseline was featureless, except for a subtle peak near 970 nm, which likely corresponds to a water (O–H) overtone band.^39^

For the 2% Pu(vi) in UNH sample, the characteristic PuO_2_^2+^ absorption band near 831 nm (^3^H_4g_ → ^3^Π_2g_ transition) was observed.^23,25^ In addition to the 831 nm peak, a broad envelope of peaks was also noted from 790 to 825 nm. Peak fitting revealed Pu(vi) peak locations near 830.7, 815.0, and 802.5 nm (Fig. S1). Relative peak heights and FWHM values are shown in Table S1. The vibrational fine structure likely corresponds to PuO_2_^2+^ vibrational levels, which arise in the solid state. Additional Pu(vi) peaks were located near 948 and 981 nm along with smaller intensity peaks from about 450 to 650 nm, also consistent with Pu(vi) spectral features in HNO_3_. The Pu(vi) spectral features were different compared to previous work performed at different Pu(vi) concentrations relative to UNH or pure Pu(vi) nitrate crystals.^6,24,25^ At 20 mol% Pu(vi) or pure Pu(vi) nitrate crystals, the Vis-NIR absorption spectrum is no longer dominated by the major transition at 831 nm but consists of multiple bands in the 775–825 nm region.

**Fig. 2:**
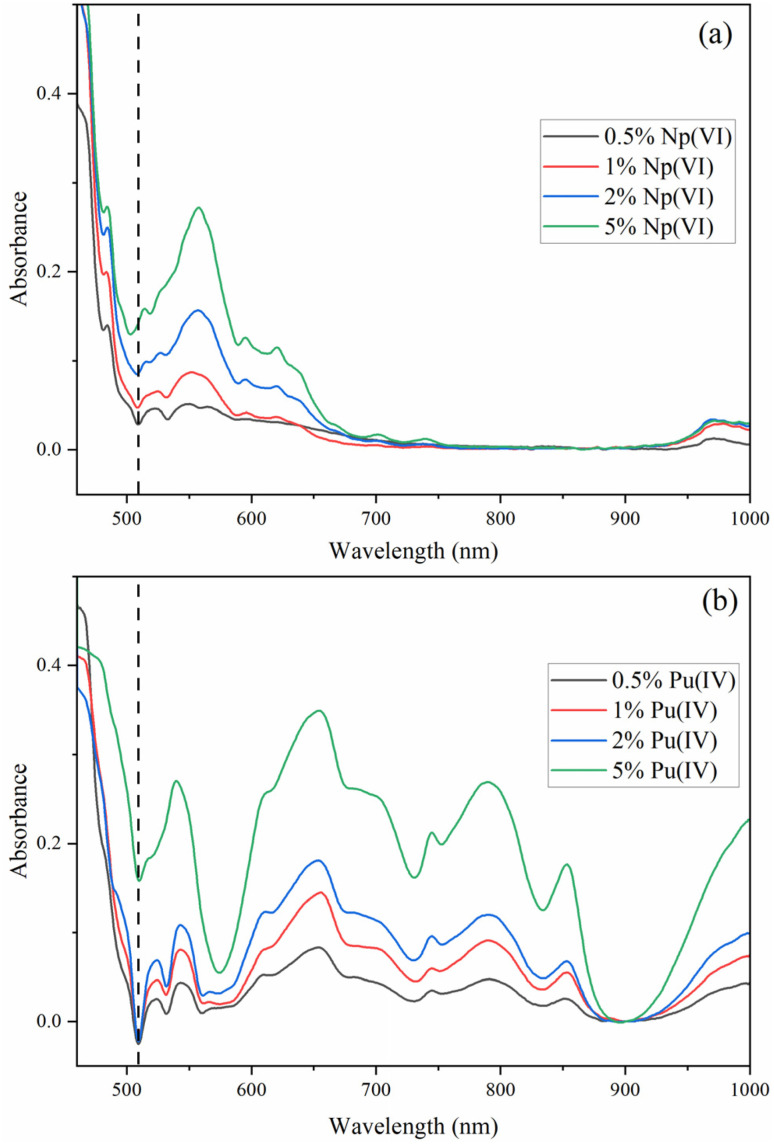
Vis-NIR reflectance spectra for (a) 0.5–5 mol% Np(vi) and (b) 0.5–5 mol% Pu(iv) crystallized with UNH. The dashed vertical line shows the location of the most intense U(vi) fluorescence peak.

Additional examples of reflectance spectra for Np(vi) and Pu(iv) are shown in Fig. 2. Characteristic Pu(iv) peaks were clearly observed in the Pu(iv) samples precipitated with UNH. The broad and overlapping peaks were more consistent with Pu(iv) solution spectra at moderate HNO_3_ concentration near the dinitrato speciation regime.^2,24^ The Np(vi) reflectance spectrum was dominated by a peak near 560 nm and additional peaks up to approximately 700 nm. The Np(vi) Vis-NIR reflectance spectra were similar to Np(vi) spectral features in HNO_3_.^40^

### U(vi) fluorescence quenching

The inverted U(vi) fluorescence peaks near 510 nm were less evident in the Pu(vi) reflectance spectrum compared to Pu(iv), indicating potential quenching of U(vi) fluorescence because of Pu(vi) incorporation into the crystal structure. With increasing Pu(vi) content, the U(vi) fluorescence band intensities decreased. Both U(vi) and Pu(vi) adopt linear dioxo geometries with similar orbital symmetries and ligand environments. The U(vi) fluorescence peaks were clearly visible in Pu(iv) samples (Fig. 1b) at the same mol% concentration as Pu(vi) samples. This finding aligns with established chemistry: Pu(iv), lacking the actinyl motif and typically exhibiting a prismatic coordination geometry, is not incorporated into the UNH lattice and thus does not quench the fluorescence of U(vi) (actinyl ion UO_2_^2+^).

The U(vi) fluorescence quenching in the Pu(iv) system was minimal, despite stronger Pu(iv) absorption bands in the U(vi) emission region than Pu(vi), which likely contributed greater inner-filter effects or competitive absorption. The phenomenon causes loss of fluorescence intensity due to absorption of the light emitted by the sample itself or the excitation light. This suggests that the Pu(vi) could act as an energy acceptor, facilitating a Förster-type energy transfer and depleting the excited-state population of U(vi) *via* vibronic coupling or excitonic migration.^41–43^ The transferred energy is dissipated non-radiatively by adjacent, non-emissive Pu(vi) centres possessing accessible low-lying excited states. A similar decrease in U(vi) fluorescence was observed when Np(vi) was co-crystallized with U(vi), consistent with their compatible energy levels and further supporting this interpretation (Fig. 2).^44,45^ Thus, U(vi) quenching is likely a function of spectral overlap as well as structural and electronic compatibility of actinyl ions within the crystal lattice of UNH.

### Principal component analysis

The relationship between absorbance and concentration in RS is typically nonlinear because the effective sample path length changes based on the total absorption coefficient of the matrix. Mixing of specular and diffuse signals, as well as sample morphology, can result in spectral shape changes between samples. Based on this and other complicated features, multivariate analysis was used to describe the system.

PCA is a useful tool for identifying sample groupings or outliers by evaluating scores and loadings plots. The X-loadings help identify which spectral variables contribute the most to each principal component (PC), and scores space can capture systematic spectral variation that correlates with the amount (*i.e.*, concentration) of a particular chemical species in each sample. PCA can also capture nonlinear trends encountered in complex spectral data using higher-order PCs. Scores were used to describe the data structure in terms of sample patterns, highlight outliers, and show differences or similarities between samples (Fig. S2).

**Fig. 3:**
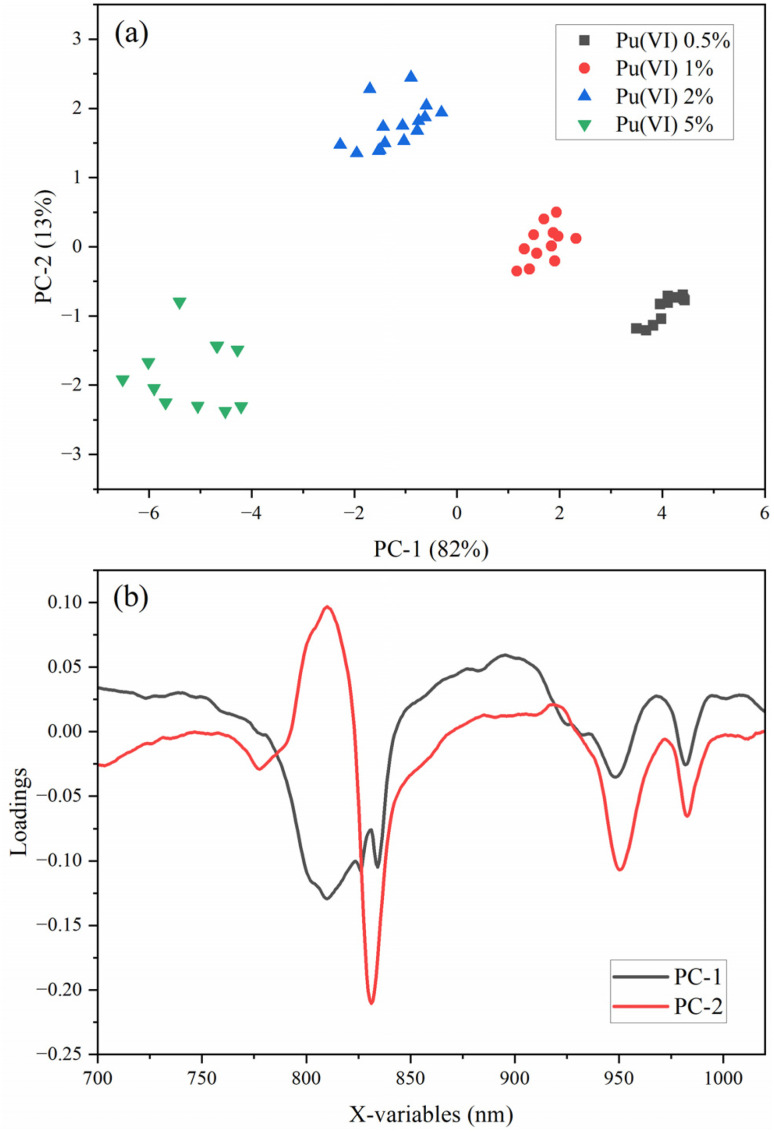
PCA model (a) 2D scores plot and (b) X-loadings for 0.5–5 mol% Pu(vi) in UNH.

PCA models were created for Pu(iv/vi) and Np(vi) to determine a relationship between concentration and spectral features. The Pu(vi) PCA model included SG filter-smoothed and SNV-treated spectra from 700 to 1030 nm. A 2D scores plot and the X-loadings for each PC for a PCA model describing the Pu(vi) concentrations is shown in Fig. 3. Distinct separation of samples along the first PC (PC1) as a function of analyte concentration was observed. The samples clustered in 2D scores space based on distinct Pu(vi) concentrations of 0.5, 1, 2, and 5 mol% relative to U. Multiple PCs were needed to capture the concentration-driven variance. PC1 described 82% of the X-variance, and the second PC (PC2) described an additional 13%. Adding a third PC (PC3) increased the total explained X-variance to 97.7%. Increasing the number of PCs beyond three did not improve the total explained X-variance.

Samples with increasing concentration levels were progressively distributed along PC1, indicating that PC1 captures the dominant variance associated with concentration. PC2 and PC3 reveal orthogonal patterns likely linked to secondary sources of variation, such as baseline shifts or matrix effects. The X-loadings plot highlights the spectral variables contributing most strongly to each PC. Key absorbance features at near 802, 814, 831, 948, and 981 nm exhibit high loadings on PC1, confirming their role in driving concentration-dependent separation. In general, absorption increased with increasing Pu concentration. Additional loading structure in PC2 suggests that the exact spectral shape had some nonlinearity from potential confounding factors at different Pu(vi) concentrations. The spread of scores in each cluster increased with increasing Pu(vi) concentration (Fig. 3). A concentration-dependent spectroscopic property is expected because increasing Pu(vi) substitution for U(vi) within the crystal lattice may influence the material properties of electronic structure and local coordination environment. At low Pu(vi) concentrations, the substitution is relatively uniform, but as the concentration increases, local distortions and symmetry breaking become more pronounced. Disorder could lead to a range of slightly different local microenvironments, which in turn cause variations in the reflectance spectrum.

Similar 2D scores plots for PCA models describing Np(vi) and Pu(iv) are shown in Fig. S3 and S4. Distinct separation of samples as a function of analyte concentration suggest that Np(vi) and Pu(iv) could also be quantified by RS and chemometric analysis. For Np(vi) and Pu(iv), PC1 described 96% and 97% of the variance, respectively. This result suggests that the features of these species are more linear than those of Pu(vi). In each actinide system, a strong correlation between the sample position along the PC1 axis and its concentration indicates that concentration is the primary source of variance in the data. The concentration of Pu(vi) appeared to influence not only band intensities but also spectral shape and peak positions. The need for multiple PCs suggests that a univariate calibration (*e.g.*, using a single wavelength) may be insufficient. Therefore, a supervised PLSR modeling approach was evaluated for Pu(vi) quantification.

### Partial least squares regression

PCA analysis does not directly relate the components to known concentrations. PLSR projects both the spectral data and the concentration values into a shared latent variable (LV) space, maximizing the covariance between predictor (spectra) and the response (concentration) matrices. PLSR is often used when overlapping bands and nonlinear effects complicate univariate calibration. The Pu(vi) Vis-NIR reflectance spectra were pre-processed using SG smoothing and SNV. Features selection helped improve performance. Only the Pu(vi) broad envelope of peaks from about 790 to 825 nm and the peaks near 948 and 981 nm were included in the model.

Replicate spectra collected from the same spot and probe orientation primarily reflect instrumental noise and short-term measurement variability rather than sample heterogeneity or scattering geometry effects. Replicate spectra of the same spot within a given sample contained minimal error (<1%). Most of the variability was due to probe positioning or the UNH sample scattering properties. Although it is reasonable to assume that the Pu(vi) distribution within the UNH crystals is homogeneous, local variations in surface texture, particle orientation, and packing density can influence light scattering and reflectance geometry. These heterogeneities, along with potential anisotropic scattering domains, may introduce spectral variability independent of chemical composition. Replicate measurements at varied locations are valuable for estimating spatial variability and assessing PLSR model robustness and precision with minimal calibration samples.^35^ The RMSER reflects noise, sample handling effects, and model stability under repeated conditions.

**Fig. 4:**
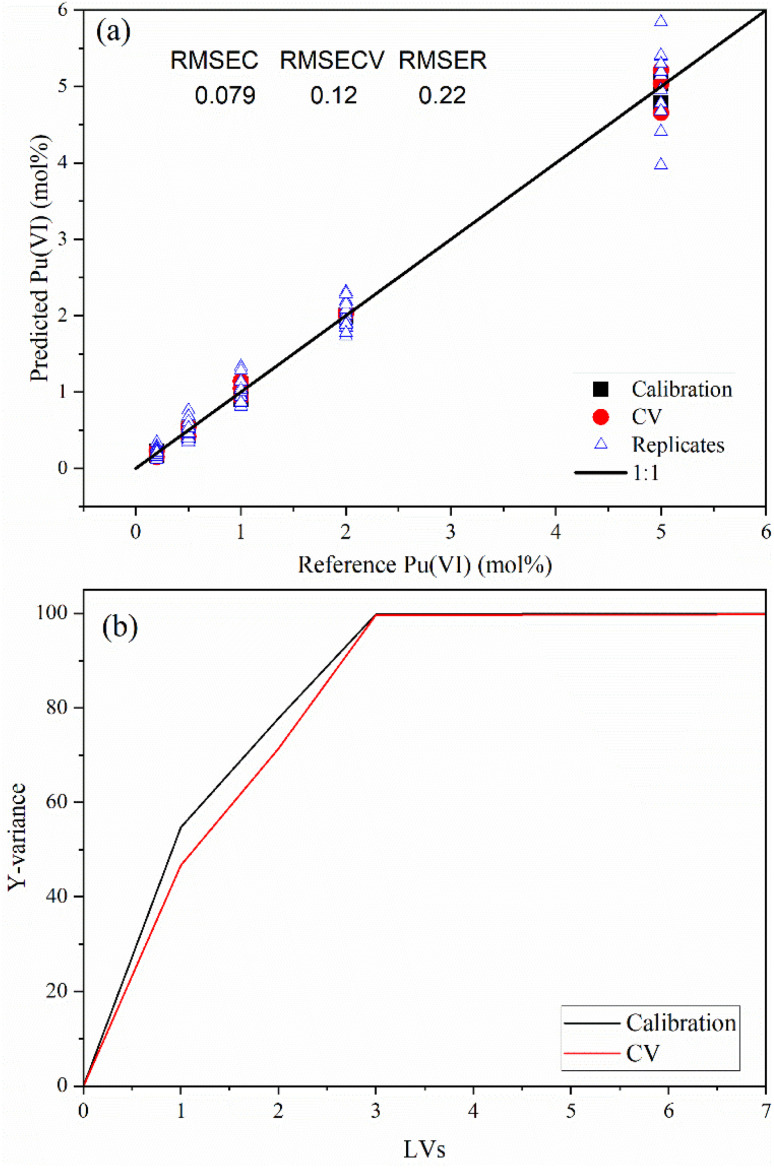
PLSR model (a) parity plot for Pu(vi) for 0.2–5 mol% Pu(vi) in UNH and (b) percent explained *Y*-variance *vs.* the number of latent variables.

PLSR model performance was visualized using a parity plot comparing predicted *versus* reference Pu(vi) concentrations for calibration, CV, and replicate measurements (Fig. 4). Calibration points aligned closely with the ideal 1 : 1 line, indicating strong model fit across the training set. Calibration and CV showed minimal deviation, supporting the model's generalizability and robustness under internal validation. Replicate measurements clustered tightly around their respective calibration values, demonstrating repeatability and low positional variance despite nonideal scattering geometries. An accompanying explained variance plot revealed that three LVs captured about 99% of the *Y*-variance in the spectral data, confirming that the model structure describes the underlying chemical information without overfitting.

Although the PLSR model was calibrated using only five concentration levels, many samples were averaged to produce triplicate spectra representative of each concentration level. By sampling multiple sample locations with varying probe positions, positional variance was captured to help minimize bias in the chemometric calibration, enhancing the reliability of remote quantification under realistic, nonideal measurement conditions. While averaging reduces spectral noise and simplifies modelling, it likely overestimates predictive accuracy by suppressing intra-sample variability. Averaging masks subtle spectral differences caused by probe repositioning, surface heterogeneity, and scattering geometry—all of which are relevant in real-world remote sensing scenarios. With the small number of calibration samples, leaving one sample out was not possible. Each sample contained vital information needed in the model. Thus, RMSEC and RMSECV values likely represent an overestimate of accuracy. The dispersion of samples observed among replicate predictions offers a more realistic estimate of precision, capturing the positional and instrumental variability that would be encountered during field deployment. The RMSER value of 0.22 suggests that a 9% error is achievable using RS. This result highlights the importance of evaluating model performance using average metrics as well as replicate-level diagnostics to ensure robustness under realistic conditions. Additional calibration samples are recommended based on the spectral complexity in the Pu(vi) system.

### Pu(vi) stability and next steps

The Pu(vi) spectra were recorded again of the five calibration samples two days and one week after crystallization. After two days, the presence of Pu(vi) was clearly noted without evidence for the ingrowth of Pu(iv). Samples still contained a majority Pu(vi) after one week; however, there was some evidence for minimal contributions from Pu(iv), particularly in the highest-concentration (5 mol%) Pu(vi) samples (Fig. S5). The PLS1 model for Pu(vi) was evaluated on the same calibration samples acquired within two days of crystallization, and the model predictions were maintained for each concentration level. The PLS1 model evaluated on the spectra collected after one week (data not shown here) showed some indication of spectral outliers in PLSR influence plots (Hotelling's T^2^*vs.* Q-residuals).^46^ Because Pu(vi) is thermodynamically unstable, future work should investigate model performance in a mixed Pu valence system with partial reduction to Pu(iv) or incomplete oxidation during crystallization.

These results demonstrate that Vis-NIR RS is effective for analysing Pu(iv/vi) and Np(vi) in solid-state UNH crystals and holds promise for broader deployment in spent nuclear fuel recycling workflows. The technique enables rapid, *in situ* quantification of Pu(vi) with bulk quantities using a reflection probe and could be adapted for micron-scale and mapping measurements.^10^ Given that Pu concentrations in used fuel typically approach 1 mol% relative to U, Vis-NIR RS is well-suited for monitoring Pu(vi) in crystallization-based reprocessing scenarios. By contrast, Np concentrations are expected to be much lower depending on the nuclear reactor and the Pu : Np ratios (*e.g.*, 30 : 1 in a light-water reactor).^47^ Based on the spectral intensities observed in this study, Np(vi) concentrations would likely fall below detection limits. Spectral overlap from Np(vi) is therefore negligible in practical applications. Potential spectral interference from impurities such as Nd(iii) should also be considered, although a rinsing step may effectively eliminate this concern.

Quantitative RS requires careful control of scattering conditions because additive and multiplicative scatter effects can obscure absorption features. Preprocessing techniques such as SNV help mitigate these effects, but consistent sample preparation remains critical. As particle size decreases, absorptivity increases while scattering becomes more pronounced. Crystals were crushed and lightly pressed by hand to minimize specular reflection and promote diffuse scattering. However, sample preparation was intentionally minimal to evaluate whether preprocessing could compensate for spectral variability to promote integration with operations-focused environments. Replicate measurements revealed positional sensitivity due to heterogeneities in surface texture, packing density, and crystal/probe orientation. These findings suggest that additional preparation steps may be necessary to improve accuracy and reproducibility. Expanding the calibration set to include more concentration levels and spatial replicates will strengthen model generalizability and support deployment in real time toward achieving remote sensing applications for process monitoring and safeguards. The ability to resolve concentration-dependent Vis-NIR absorption features for Pu and Np in UNH crystals represents a significant advancement toward developing remote monitoring tools to support crystallization-based reprocessing.

## Conclusions

The Pu(iv/vi) reflectance spectra in UNH contain features that can be leveraged for the quantification of Pu. The observed quenching of U(vi) fluorescence upon co-crystallization with Pu(vi) is attributed to lattice-mediated electronic coupling between structurally analogous actinyl species. Complicated crystal structure properties and geometrical considerations lead to significant variation in the reflectance spectra, particularly for Pu(vi). Despite this spectral complexity, preprocessing and chemometric modelling was leveraged to quantify Pu(vi) in UNH by PLSR. The stability of Pu(vi) in the UNH matrix was confirmed for at least one week. To improve PLSR model robustness, further refinement of sample preparation protocols is recommended, including controlled particle size reduction and surface homogenization to minimize specular reflection and scattering variability. Future work should expand the reflectance spectroscopy framework to accommodate more-complex sample matrices and operational conditions relevant to nuclear reprocessing. Overall, chemometric analysis offers a useful approach for using RS to quantify Pu(vi) in UNH. The ability to detect concentration-dependent Vis-NIR absorption spectroscopic changes for Pu and Np in uranyl nitrate crystals is significant for the development of monitoring capabilities to support a crystallization reprocessing technique.^5,48,49^ This approach greatly expands the applicability of optical spectroscopy for the remote quantification of Pu in complex solid-state matrices.

## Author contributions

The manuscript was written using contributions of all authors. All authors have given approval to the final version of the manuscript.

## Conflicts of interest

The authors declare no competing financial interest.

## Supplementary Material

RA-015-D5RA08550K-s001

## Data Availability

The datasets generated during and/or analyzed during the current study are available from the corresponding author on reasonable request. Supplementary information (SI) is available. See DOI: https://doi.org/10.1039/d5ra08550k.
