# Supplementary material for: Remote sensing of Pu in uranyl nitrate crystals using reflectance spectroscopy and chemometrics
Source: RSC Adv. 2025 Dec 9;15(57):48807–15. doi: 10.1039/d5ra08550k (PMC12687128; doi:10.1039/d5ra08550k)
Supplement: RA-015-D5RA08550K-s001 [file RA-015-D5RA08550K-s001.pdf]

Supplementary Information (SI) for RSC Advances.
This journal is © The Royal Society of Chemistry 2025

Supporting information

Remote Sensing of Pu in Uranyl Nitrate Crystals Using Reflectance Spectroscopy and Chemometrics

Luke R. Sadergaski[1], Jeffrey D. Einkauf[1], Laetitia H. Delmau[1], Jonathon D. Burns[2]

[1]Oak Ridge National Laboratory, Oak Ridge, Tennessee, USA
[2]Department of Chemistry, University of Alabama at Birmingham, Birmingham, AL, United States

This document contains:

Tables: 1
Figures: 5
Pages: 4

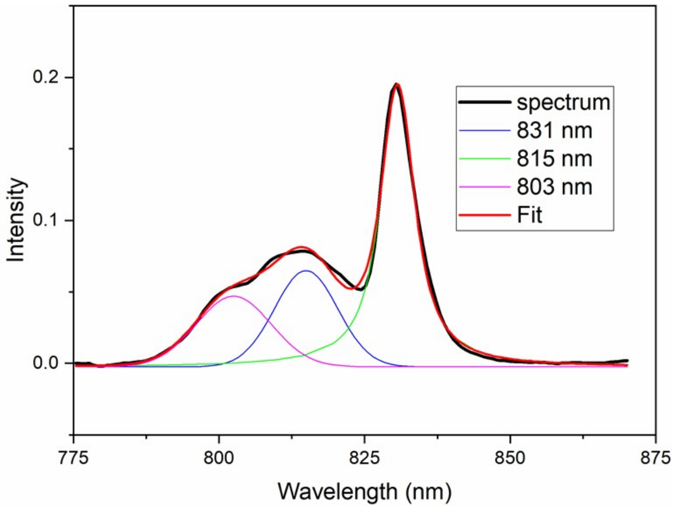

Fig. S1. Voigt peak fit analysis of 2 mol % Pu(VI) in UNH. Peaks were located near 830.7, 815.0, and 802.5 nm. R^2 value is 0.995.

Table S1. Max height and FWHM for Pu(VI) peaks shown in Fig. S1.

Peak	Height	FWHM
802.5	0.049	7.1
815.0	0.067	12.8
830.7	0.20	15.1

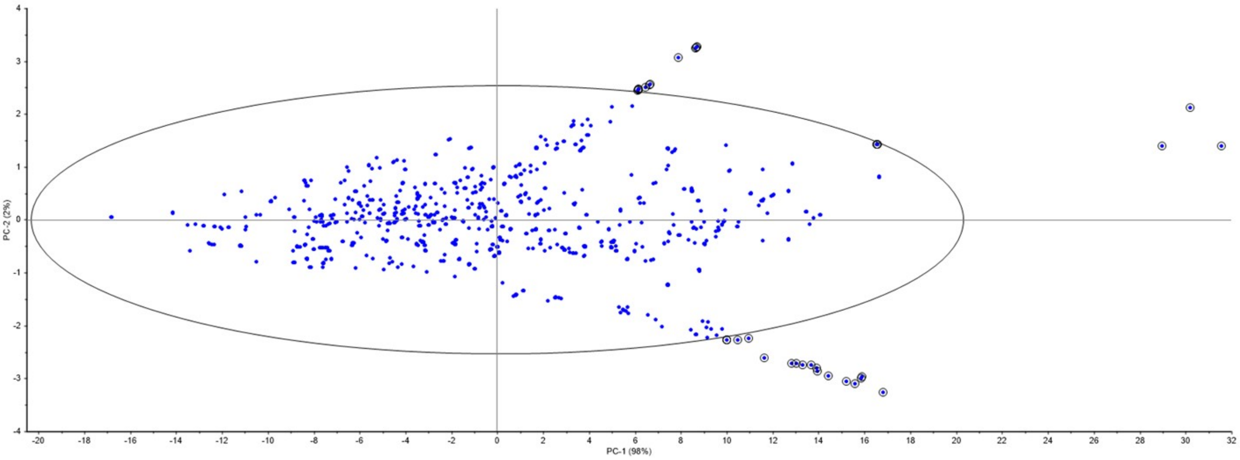

Fig. S2. 2D scores plots of PC1 versus PC2 with outliers marked outside the 99% Hotelling's T^2 confidence ellipse.

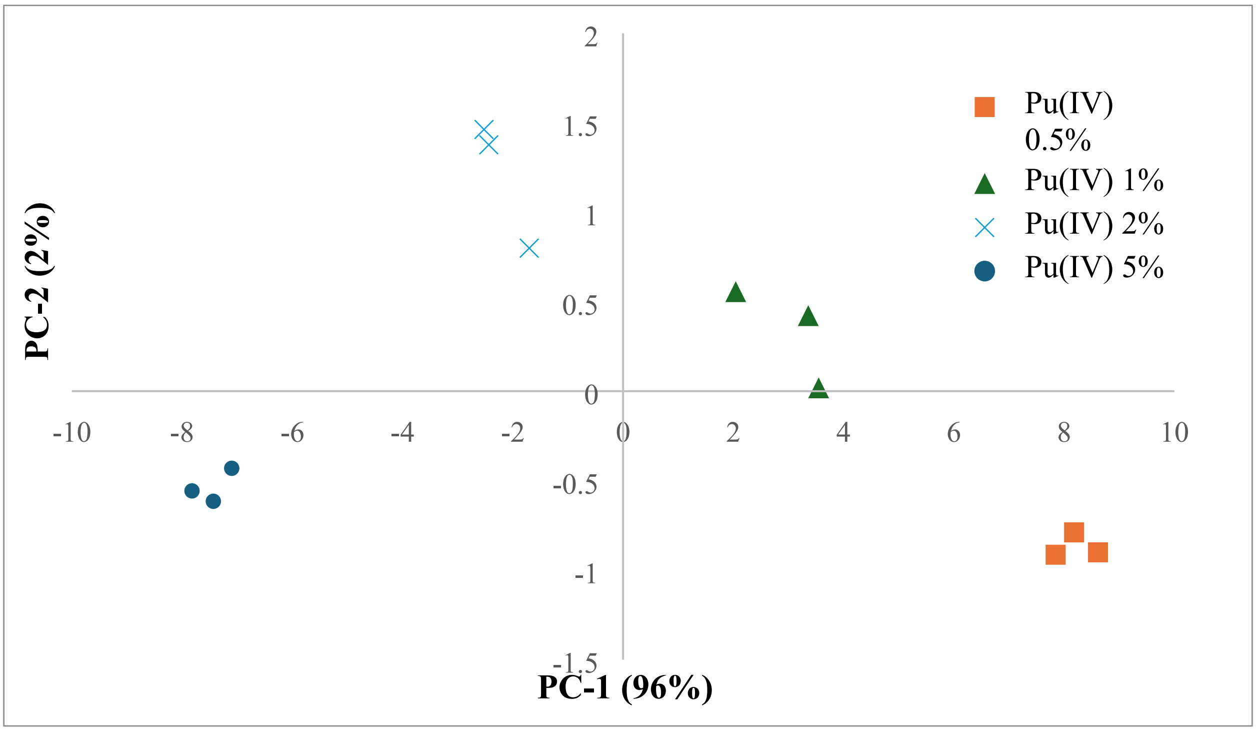

Fig. S3. PCA model 2D scores plot for triplicate Pu(IV) spectra showing groups by Pu(IV) concentration.

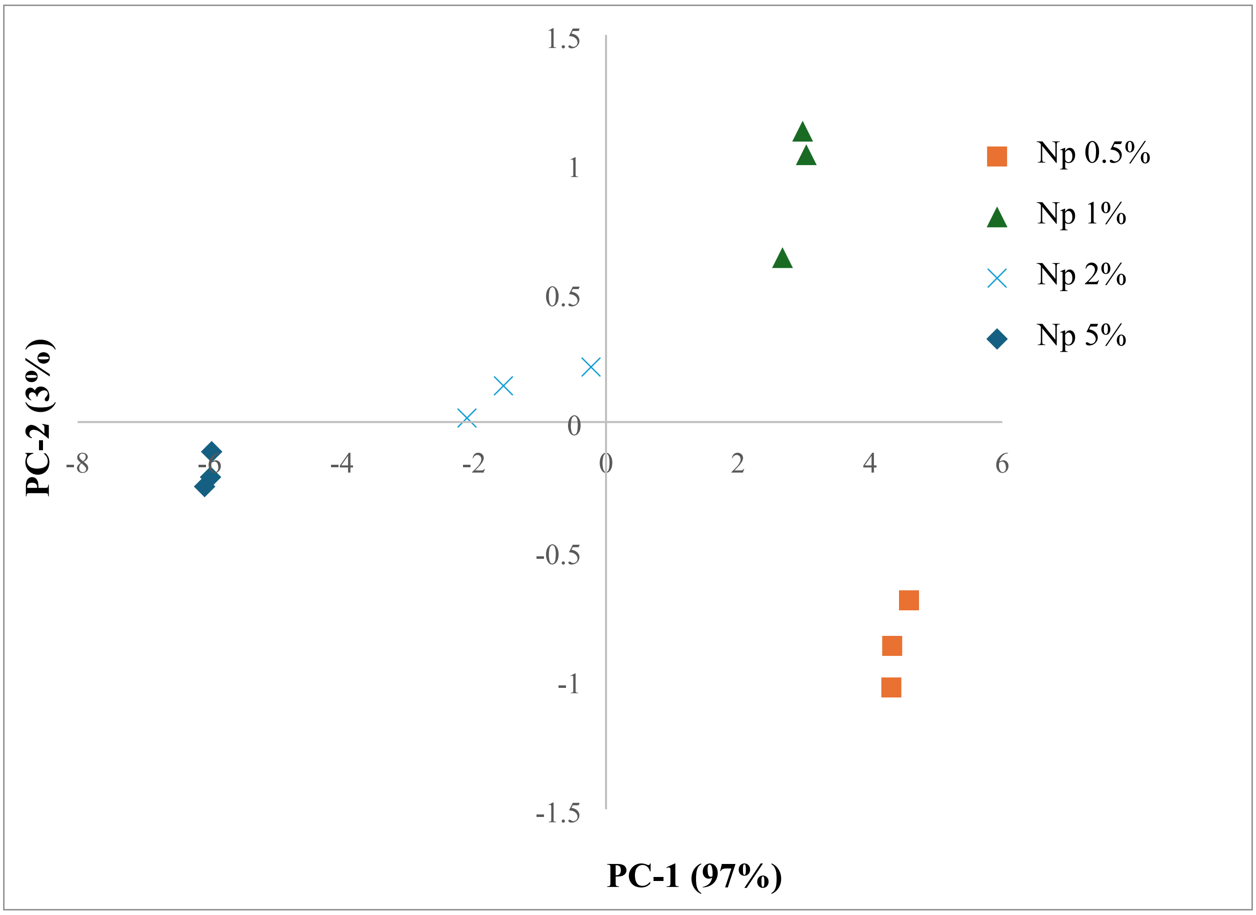

Fig. S4. 2D scores plot for triplicate Np(VI) spectra showing groups by Np(VI) concentration.

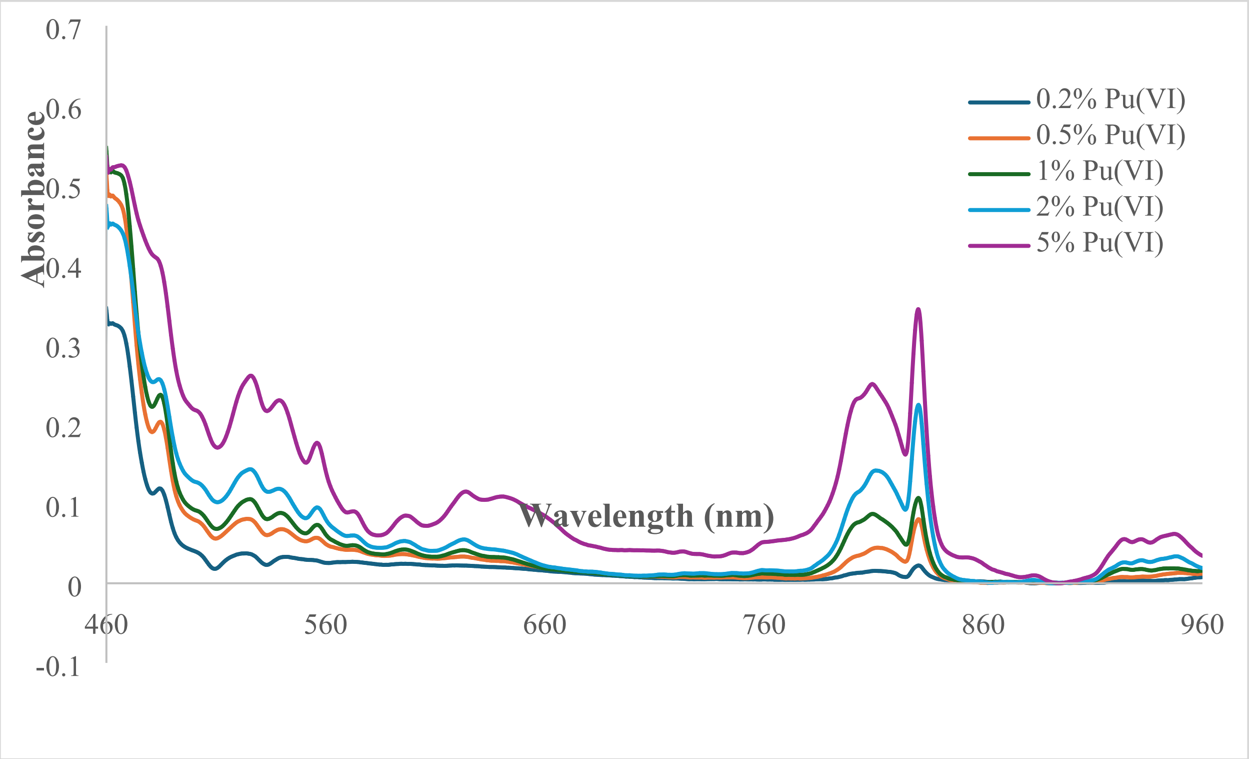

Fig. S5. Diffuse reflectance spectra of Pu(VI) in UNH one week after crystallization. Subtle peaks near 645 and 858 nm as well as distortion near 928 nm indicate potential Pu(IV).

Justification:

Specular (or regular) reflections are associated with mirror-like surfaces, and diffuse reflection results from opaque surfaces often encountered with powders. Reflectance is preferred over transmission techniques if the sample cannot be pressed into a KBr pellet or the beam of light cannot pass through the sample.
